# CCR7-dependent trafficking of RORγ^+^ ILCs creates a unique microenvironment within mucosal draining lymph nodes

**DOI:** 10.1038/ncomms6862

**Published:** 2015-01-09

**Authors:** Emma C. Mackley, Stephanie Houston, Clare L. Marriott, Emily E. Halford, Beth Lucas, Vuk Cerovic, Kara J. Filbey, Rick M. Maizels, Matthew R. Hepworth, Gregory F. Sonnenberg, Simon Milling, David R. Withers

**Affiliations:** 1 MRC Centre for Immune Regulation, Institute for Biomedical Research, College of Medical and Dental Sciences, University of Birmingham, Birmingham B15 2TT, UK; 2 Institute of Infection, Immunity and Inflammation, University of Glasgow, Glasgow G12 8TA, UK; 3 Institute of Immunology and Infection Research, University of Edinburgh, Edinburgh EH9 3FL, UK; 4 Division of Gastroenterology, Joan and Sanford I. Weill Department of Medicine, New York, New York 10021, USA; 5 The Jill Robert’s Institute for Research in Inflammatory Bowel Disease, Weill Cornell Medical College, New York, New York 10021, USA; 6 Department of Microbiology and Immunology, The Jill Robert's Institute for Research in Inflammatory Bowel Disease, Weill Cornell Medical College, New York, New York 10021, USA

## Abstract

Presentation of peptide:MHCII by RORγ-expressing group 3 innate lymphoid cells (ILC3s), which are enriched within gut tissue, is required for control of CD4 T-cell responses to commensal bacteria. It is not known whether ILC populations migrate from their mucosal and peripheral sites to local draining secondary lymphoid tissues. Here we demonstrate that ILC3s reside within the interfollicular areas of mucosal draining lymph nodes, forming a distinct microenvironment not observed in peripheral lymph nodes. By photoconverting intestinal cells in *Kaede* mice we reveal constitutive trafficking of ILCs from the intestine to the draining mesenteric lymph nodes, which specifically for the LTi-like ILC3s was CCR7-dependent. Thus, ILC populations traffic to draining lymph nodes using different mechanisms.

## Abstract (web summary)

Innate lymphoid cells have an important role in mucosal immunity and present peptide:MHCII to CD4 T cells. Here the authors show that innate lymphoid cell subsets migrate from the gut mucosa to the draining lymph nodes via different mechanisms, where they form distinct microenvironments.

## Graphical abstract

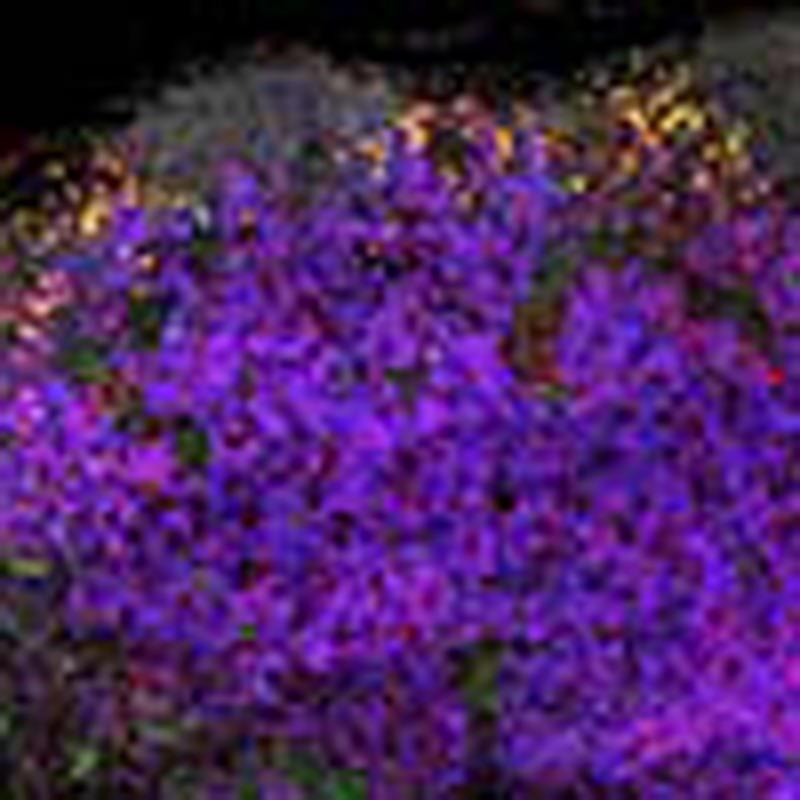

Secondary lymphoid tissues, such as lymph nodes (LNs), provide a highly organized microenvironment that promotes the chance encounter of antigen-specific lymphocytes with their cognate antigen. The system of lymphatic vessels that connects these structures allows immune cells and antigen to drain from sites of infection, resulting in an efficient adaptive immune response to immunological challenge. The formation of LNs is dependent upon the correspondingly named lymphoid tissue inducer (LTi) cell, a retinoic acid receptor-related orphan receptor γt (RORγt)-dependent population in the embryo that provides the critical lymphotoxin signals to developing stromal cells^1^,^2^,^3^,^4^. LTi cells are now described among the group 3 innate lymphoid cells (ILC3) of the ILC family^5^. Within adult mice, LTi-like cells persist^6^,^7^, alongside NKp46^+^ ILC3s^8^ and colitogenic NKp46^−^ ILC3s (that appear distinct from LTi-like cells)^9^. All of these ILC3s are thought to be the key cytokine producers within the intestine, aiding epithelial barrier integrity through the production of IL-22, although they may also drive intestinal inflammation^9^. Within the gut, ILC3s are also required for development of isolated lymphoid follicles and T-cell-independent switching to IgA^10^,^11^. Consistent with a role in CD4 T-cell responses, only the LTi-like cells found in the adult express co-stimulatory molecules such as OX40L and CD30L, associated with CD4 T-cell survival^6^,^12^ and indicating distinct functions in the developed immune system. Furthermore, the absence of RORγ-expressing cells resulted in impaired memory CD4 T-cell survival^13^. Crucially, it was recently demonstrated that LTi-like ILC3s can present antigen in the context of major histocompatibility complex II (MHCII)^14^, and this was required for normal regulation of CD4 T-cell responses to commensal bacteria. The mechanisms by which ILC3s regulate CD4 T-cell responses and the site where this occurs are unknown. How, for example, do the ILC populations in secondary lymphoid tissue relate to those in the periphery? It is possible that specific ILC subsets migrate to secondary lymphoid tissue in order to present peptides, akin to dendritic cells (DCs).

To better understand this, here we investigate the ILC composition of a range of LNs, comparing those that drain peripheral tissues to those that drain mucosal sites. While ILCs can be detected in all LNs analysed, RORγ^+^ ILC3s are enriched within mesenteric (m) and mediastinal (md) LNs, which drain mucosal tissues. Within the mLNs, ILC3s reside exclusively within the interfollicular spaces, where they sit within close proximity to GATA-3^+^ ILC2s and form a microenvironment that was not detected within inguinal (i), brachial (b) or popliteal (p) LNs. Studies with Kaede transgenic mice demonstrate constitutive trafficking of ILCs from the gut to the mLN. Migration of LTi-like ILC3s, but not other ILC subsets, is dependent upon CCR7. Therefore, our data reveal that ILC populations utilize different mechanisms to traffic to secondary lymphoid tissue, with mucosal-draining LNs containing a distinct interfollicular microenvironment populated by LTi-like ILC3s.

## Results

### ILC3s are the main ILC group in mucosal-draining LNs

**Figure 1:**
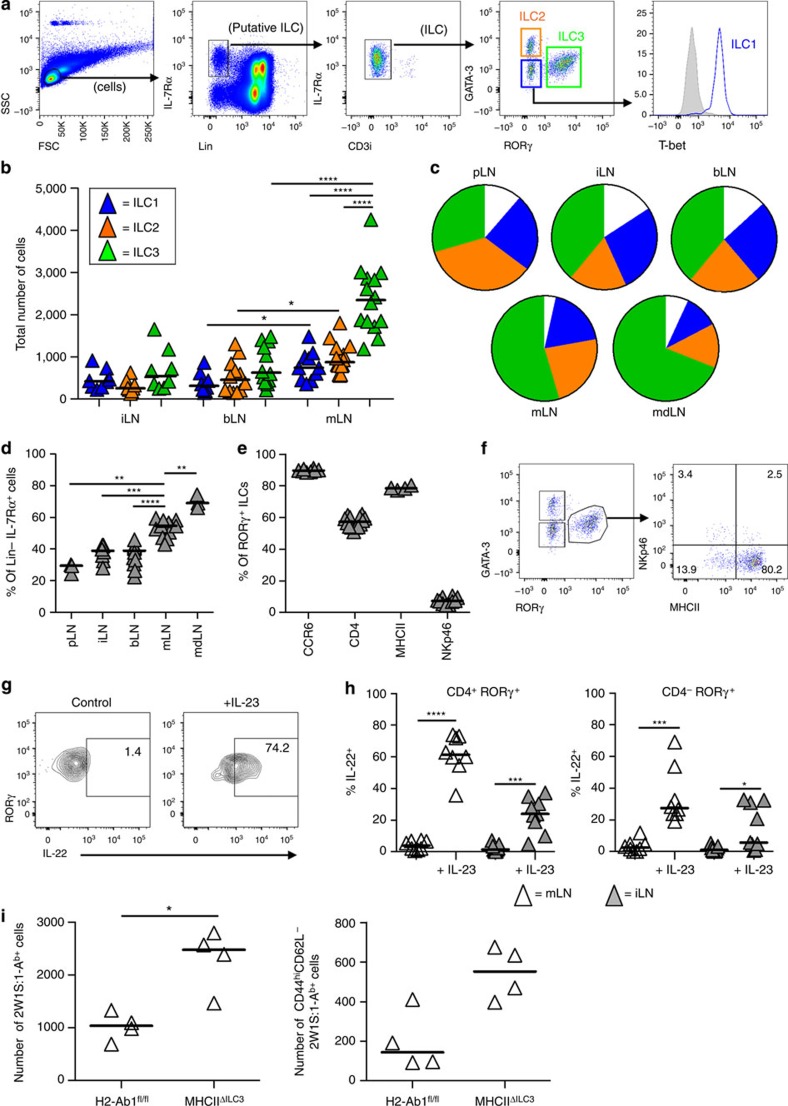
ILC composition of mucosal LNs differs from peripheral LNs. (**a**) Gating strategy for detection of ILC populations by flow cytometry with T-bet^+^ ILC1, GATA-3^+^ ILC2 and RORγ^+^ ILC3. T-bet isotype control is shown in grey. Plots are representative of 10 mice from three independent experiments. (**b**) Enumeration of ILC1, ILC2 and ILC3 cells per individual inguinal (i; *n*=8), brachial (b; *n*=12 except for ILC1 where=8) or mesenteric (m; *n*=14 except for ILC1 where=10) LNs of WT mice. Data pooled from a minimum of three independent experiments. (**c**) Pie charts showing the median percentage of total Lin^−^IL-7Rα^+^ cells that are ILC1, ILC2 and ILC3 populations in different LNs, including also popliteal (p; *n*=3) and mediastinal (md; *n*=3) LNs. Data pooled from a minimum of three independent experiments; white segment shows % cells outside transcription factor gates. (**d**) Percentage of ILCs expressing RORγ in different LNs (*n*=3, 8, 12, 14, 3 mice per group), pooled from a minimum of three independent experiments. (**e**) Percentage of RORγ^+^ ILC3s expressing CCR6, CD4, MHCII and NKp46 in mLN. (**f**) Expression of MHCII and NKp46 by RORγ^+^ ILC3s within the mLN. Plots are representative of 10 mice from three independent experiments. (**g**) Expression of IL-22 by CD4^+^ RORγ^+^ ILC3s. Plots are representative of eight mice from three independent experiments. (**h**) Percentage of CD4^+^ and CD4^−^ RORγ^+^ ILC3s in mLN and iLN expressing IL-22 after culture with IL-23 (*n*=8, 9). Data pooled from three independent experiments. (**i**) Number of 2W1S:I-A^b+^ CD4 T cells per peripheral LN pool from *H2-Ab1*^*fl*/*fl*^ and *MHCII*^Δ*ILC3*^ mice (*n*=4, 4) 6 days post i.p. immunization with 2W1S peptide. Data pooled from one experiment. *P<0.05, **P<0.01, ***P<0.001 and ****P<0.0001 (Mann–Whitney non-parametric, two-tailed test). Bars represent the median values in all graphs.

Many studies of ILCs have focused on their role at barrier sites of the body such as the gastrointestinal tract, the lung and the skin^9^,^15^,^16^,^17^,^18^,^19^,^20^. Although ILC populations have been described within the secondary lymphoid tissue^9^,^12^,^14^,^17^,^20^,^21^, this has often been in mice lacking B and T cells^9^,^12^,^20^. Therefore, initially we sought to analyse the ILC populations present within LNs that drain distinct anatomical sites in wild-type (WT) mice. Given concerns that surface markers such as Thy1 are not definitive, we first identified IL-7Rα^+^Lin (B220, CD3, CD5 and CD11c)^−^ cells, and then used the expression of T-bet, GATA-3 and RORγ to identify the ILC1, ILC2 and ILC3 groups, respectively (Fig. 1a). This strategy excludes conventional natural killer (NK) cells and focuses the analysis to the ‘helper’ ILC subsets recently shown to derive from an Id2^+^IL-7Rα^+^ progenitor^22^. Intracellular staining for CD3 was included to ensure exclusion of T cells. Per bLN or iLN, comparable numbers of ILC1s, ILC2s and ILC3s were detected (Fig. 1b). Notably, the number of ILC1s, ILC2s and ILC3s per individual mLN was significantly higher than in the peripheral-draining LNs (Fig. 1b), and within mLNs there were significantly more ILC3s than any other type of ILC, notable since these nodes drain the ILC3-enriched intestinal tissue^10^,^16^. Analysis of the ILC composition of each LN, shown as % of the IL-7Rα^+^Lin^−^ population, further highlighted the dominance of the ILC3 population within the mLN, although ILC3s were still present in all the peripheral LNs assessed (Fig. 1c,d). Surprisingly, the mdLN ILC populations were also dominated by ILC3s (Fig. 1c,d), despite the strong association of ILC2s with the lung and the lack of ILC3s in lung tissue^16^,^23^,^24^. In contrast, the proportion of ILC3s was lowest within the pLN, a small peripheral LN (Fig. 1c,d), revealing an ILC3 bias within mucosal tissue-draining LNs. In the gut, ILC3s can be subdivided based on cytokine expression and expression of the surface markers CCR6, CD4, MHCII and NKp46 (refs 9, 18, 25). We found that the majority of ILC3s in the mLN (Fig. 1e,f) were CCR6^+^CD4^+/−^MHCII^+^NKp46^−^, consistent with the previously described LTi-like population^26^. Although a minor population of NKp46^+^ ILC3s were also detected, the CCR6^−^CD4^−^RORγ^+^T-bet^+^ population detected in the gut^25^ was largely absent from WT mLNs. Within the ILC1 group, a population of ex-ILC3 cells has been described, where expression of RORγ is lost and T-bet and NKp46 expression is turned on. Using *RORγ cre* x *ROSA26 eYFP* fate mapping mice (Supplementary Fig. 1) we found that in all LNs analysed, a substantial population of T-bet^+^eYFP^+^RORγ^−^ cells were evident. The ability of the LN ILC3s to produce IL-22 was confirmed by *in vitro* culture with IL-23 (Fig. 1g), with ILC3s in the mLN significantly better at producing IL-22 than their counterparts in iLNs (Fig. 1h).

Since ILC3s regulate CD4 T-cell responses to commensal bacteria^14^, we sought to further assess whether ILC3s limit CD4 T-cell responses within LNs following immunization. Mice in which ILC3s lack MHCII (*MHCII*^Δ*ILC3*^)^14^ were immunized intraperitoneally (i.p.) with 2W1S peptide and the endogenous CD4 T-cell response assessed in a pool of ILC3-containing peripheral LNs. Within the peripheral LNs of *MHCII*^Δ*ILC3*^ mice 6 days post immunization, the 2W1S response was substantially enhanced compared with controls and 2W1S-specific CD4 T cells showed increased frequencies of cells with an effector phenotype (CD44^+^CD62L^lo^; Fig. 1i), consistent with a regulatory function for ILC3s in limiting CD4 T-cell responses through an MHCII-dependent mechanism.

### ILC3s form a distinct microenvironment in mucosal LNs

**Figure 2:**
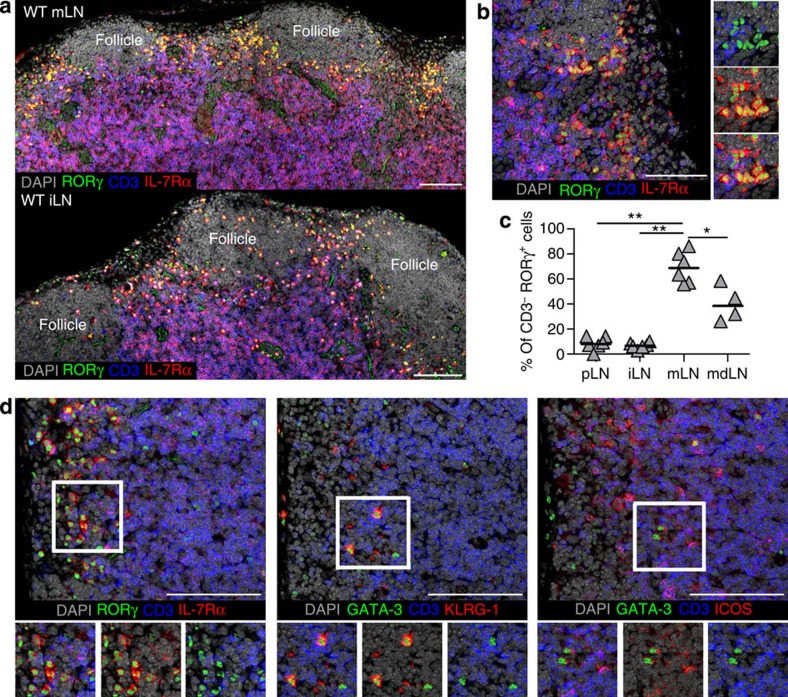
ILC2 and ILC3 cells reside within the interfollicular spaces. (**a**) Tilescanned images of murine mLN and iLN stained for expression of RORγ, CD3 and IL-7Rα, counterstained with DAPI. (**b**) Magnified image showing interfollicular area of mLN, stained for expression of RORγ, CD3 and IL-7Rα, counterstained with DAPI. Small panels show numerous RORγ^+^CD3^−^IL-7Rα^+^ cells. (**c**) Quantitation of RORγ-expressing cells within the interfollicular areas of different LNs, showing percentage of RORγ^+^ cells lacking CD3 expression (*n*=6, 6, 6, 4 areas from three WT mice). *P<0.05, **P<0.01 (Mann–Whitney non-parametric, two-tailed test) bars represent the median values. (**d**) Serial sections of mLN showing the same interfollicular area stained for ILC3s (RORγ^+^CD3^−^IL-7Rα^+^) and ILC2s (GATA-3^+^CD3^−^KLRG-1^+^ or GATA-3^+^CD3^−^ICOS^+^). Scale bar in all images represents 100 μm. Data representative of at least three WT mice per stain per tissue.

Immunofluorescence staining was used to assess the location of ILC3s within LNs. Cells identified as ILC3s (RORγ^+^IL-7Rα^+^CD3^−^) (Fig. 2a,b) were located almost exclusively within the interfollicular spaces of the mLN, with a scattering of cells further into the T zone at the interface with the follicle (Fig. 2a,b). Besides ILC3s, T cells expressing RORγ (RORγ^+^IL-7Rα^+^CD3^+^) were identified within the interfollicular areas and T zone. To quantify ILC3s and RORγ^+^ T cells within the interfollicular areas, regions between B-cell follicles were marked and the percentage of RORγ^+^IL-7Rα^+^ cells lacking CD3 calculated (Fig. 2c and Supplementary Fig. 2). These data demonstrate that the interfollicular areas of the mLN are dominated by ILC3s, while in the iLN and pLN most cells expressing RORγ are T cells, consistent with the flow cytometric analysis detecting fewer ILC3s within these tissues.

Given their distinctive location, we also investigated whether ILCs are important for maintaining stromal cell populations within these tissues, a role that would be analogous to ILC3 function in the embryo. Different stromal cells were analysed in sections of LN from bone marrow (BM) chimeras where ILC3s were heavily depleted. No gross effects were evident in LN architecture with normal segregation of B and T cell areas. Lymphatic vessels and high endothelial venules appeared comparable between depleted and control chimeras (Supplementary Fig. 3). The marginal reticular cell population, identified by expression of RANKL and located at the outer edge of follicles and thus in close proximity to ILCs also appeared normal (Supplementary Fig. 3). Therefore no overt changes in LN structure or the composition of LN stromal populations were evident after ILC3 depletion.

The location of ILC2s within secondary lymphoid tissue has not yet been described. To identify ILC2s within mLN sections, expression of GATA-3 and ICOS or GATA-3 and KLRG-1 (ref. 27) were assessed. Cells lacking CD3 and expressing either GATA-3 and KLRG-1 (ref. 27) or GATA-3 and ICOS^17^ were identified within the interfollicular spaces alongside ILC3s (Fig. 2d). Although fewer in number (Fig. 1b), this suggests that both ILC2s and ILC3s reside within close proximity within a LN.

Given that T cells expressing RORγ reside in similar areas in the LN to ILC3s, we also sought to understand the contribution that ILC3s make to the total RORγ^+^ population (Supplementary Fig. 4). Gating on RORγ^+^B220^−^CD11b^−^CD11c^−^cells, we found that ILC3s constituted a very minor (<5%) proportion of RORγ^+^ cells in peripheral LNs; however, a significantly larger part of the RORγ^+^ population in mLNs (>20%). Collectively, our data reveal that ILC3s are more numerous in LNs draining mucosal tissue, and that they form a distinct microenvironment within the interfollicular spaces.

### Altered ILC frequencies in lymphopenic mice

**Figure 3:**
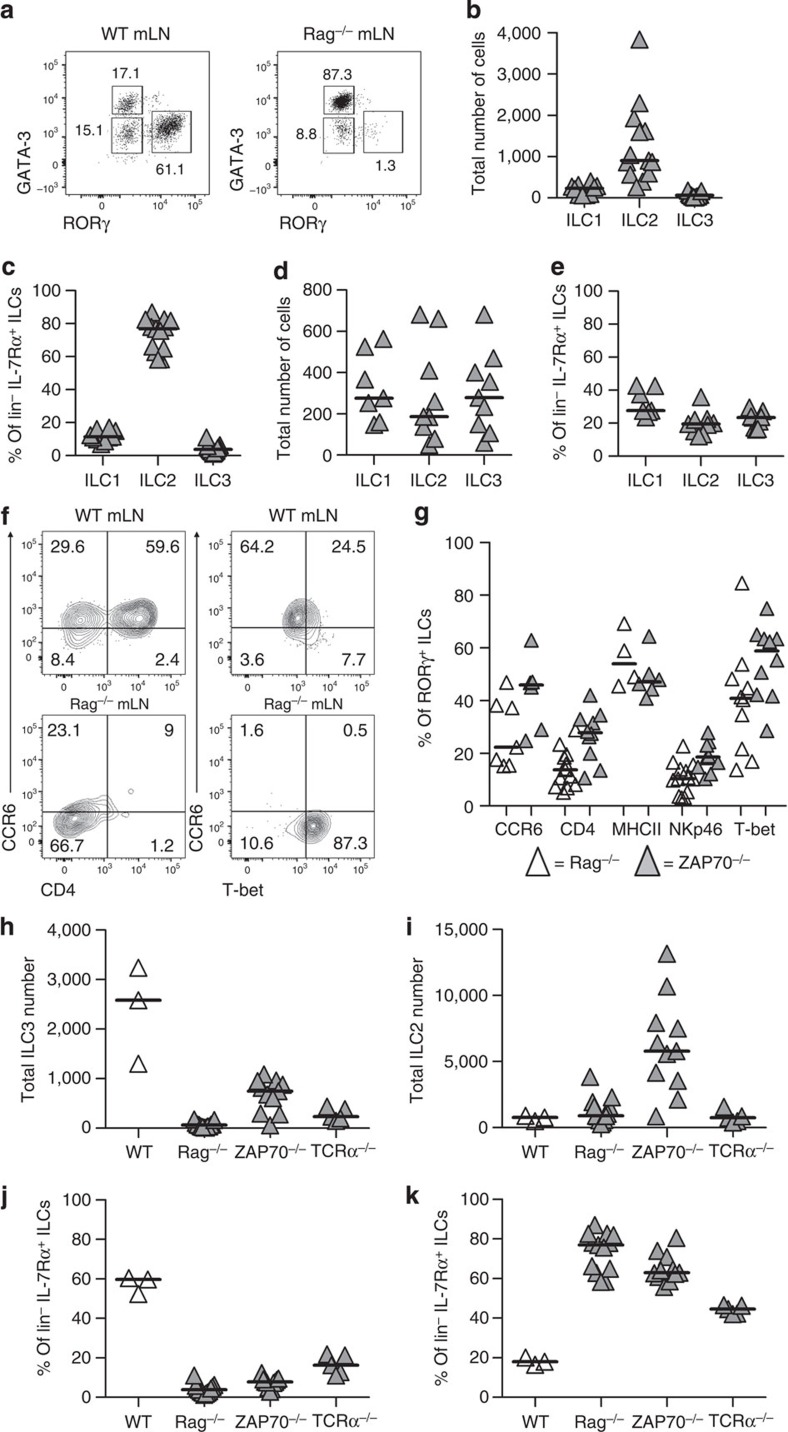
Altered ILC populations in the mLN of lymphopenic mice. (**a**) Detection of ILC populations with flow cytometry in the mLN of WT and *Rag*^−/−^ mice. (**b**) Enumeration and (**c**) percentage of ILC1, ILC2 and ILC3 cells in mLN pool of *Rag*^−/−^ mice (*n*=9, 12, 12 in **b**, 10, 13, 13 in **c**). (**d**) Enumeration and (**e**) percentage of ILC1, ILC2 and ILC3 cells in *Rag*^−/−^ iLN (*n*=7, 9, 9). (**f**) Example of CCR6 and CD4 or T-bet staining on RORγ^+^ ILCs in mLN of WT and *Rag*^−/−^ mice. (**g**) Percentage of RORγ^+^ ILC3s expressing CCR6, CD4, MHCII, NKp46 and T-bet in *Rag*^−/−^ and *ZAP70*^−/−^ mLN (*n*=7, 6, 13, 10, 4, 6, 13, 10, 10 and 10), pooled from minimum of two independent experiments except MHCII, which is 1. Number of ILC3 (**h**) and ILC2 (**i**) cells per individual mLN from WT, *Rag*^−/−^, *ZAP70*^−/−^ and *TCRα*^−/−^ mice (*n*=3, 12, 11 and 5). Percentage of ILC population with ILC3 (**j**) and ILC2 (**k**) phenotype in mLN from WT, *Rag*^−/−^ *ZAP70*^−/−^ and *TCRα*^−/−^ mice (*n*=3, 12, 11 and 5). Data pooled from minimum of two independent experiments. Bars represent the median values in all graphs.

Studies of ILC3 function have often relied upon *Rag*^−/−^ mice and anti-Thy1 antibody (Ab) treatment to deplete these cells *in vivo*^9^,^20^. To enable comparison of our data with previously published studies, we sought to assess the ILC composition of LNs in *Rag*^−/−^ mice using the flow cytometric analysis described above (Fig. 3a). Surprisingly, within the mLN, this differed vastly from that of WT mice, with GATA-3^+^ ILC2s replacing RORγ^+^ ILCs as the dominant population (Fig. 3a–c). Furthermore, this difference was mLN-specific, since within iLNs a comparable percentage of each ILC type was detected (Fig. 3d,e). The majority of the ILC3s in *Rag*^−/−^ mLNs lacked CCR6 and CD4 expression (Fig. 3f,g), thus resembling a distinct population described within the intestine^25^, which is not detected in WT mLN (Fig. 1e,f). Given the disrupted architecture within *Rag*^−/−^ LNs, mLNs from other T-cell-deficient mice were also assessed with similar results (Fig. 3g–k). Therefore, the composition of ILCs within the gut-draining mLN is altered in T-cell-deficient mice highlighting the importance of using WT mice. In addition, the high frequency of ILC2 cells within T-cell-deficient mLNs enabled us to confirm our previous location studies. Within *ZAP70*^−/−^ mLN, as in WT, ILC2s were readily detected within the interfollicular spaces and at the B:T interface, interspersed with the ILC3s (Supplementary Fig. 5).

### ILCs traffic from the gut to the mLN

**Figure 4:**
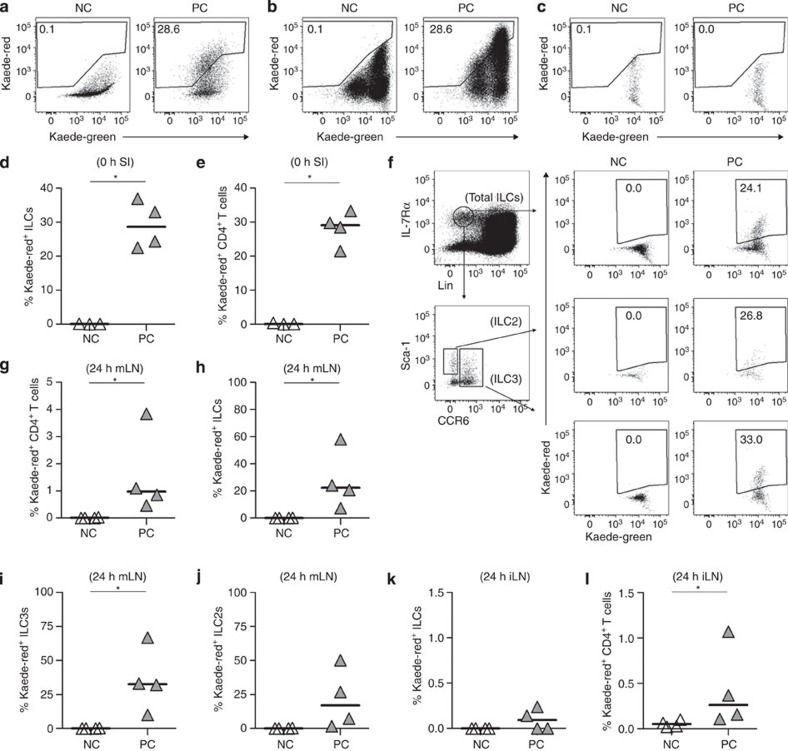
Trafficking of ILCs from the intestine to the mLN. To assess trafficking of ILCs from the intestine to the mLN, the small intestines of *Kaede* mice were exposed to violet light after surgical exposure. At each time point expression of Kaede-red was assessed through comparing tissues of mice exposed to violet light with those of ambient light-exposed surgery controls. Expression of Kaede-red by ILCs (**a**) and CD4 T cells (**b**) in the small intestine at 0 h post surgery. (**c**) Expression of Kaede-red by ILCs in the mLN at 0 h post surgery. Percentage of ILCs (**d**) and CD4 T cells (**e**) expressing Kaede-red protein at 0 h post surgery (*n*=3 and 4). (**f**) Expression of Kaede-red by total ILCs, Sca-1^+^ putative ILC2s and CCR6^+^ ILC3s in the mLN ~24 h post surgery, here using stated cell surface markers to define ILC populations in absence of transcription factor staining. Percentage of CD4 T cells (**g**), ILCs (**h**), ILC3s (**i**) and ILC2s (**j**) expressing Kaede-red protein in the mLN at 24 h post surgery, (*n*=4 and 4). Percentage of ILCs (**k**), CD4 T cells (**l**), expressing Kaede-red protein in the iLN at 24 h post surgery (*n*=4, 4). *P<0.05 (Mann–Whitney non-parametric, two-tailed test, except **d**,**e** where unpaired *t*-test was used). Data representative of three independent experiments, bars represent the median values in all graphs. SI, small intestine, NC, non-converted ambient light-exposed surgery controls, PC, photoconverted mice exposed to violet light.

Since ILC3s are concentrated within the gut, we sought to test whether an ILC3 bias in the mLN reflected direct trafficking of ILC3s from the intestine to the mLN using transgenic *Kaede* mice^28^. Here cells express the photoconvertible Kaede protein that converts irreversibly from green to red fluorescence following violet light exposure. Following laparotomy via a small midline incision, the small intestines of *Kaede* mice were carefully exposed to violet light (labelled ‘photoconverted’, or PC), while the mLN and Peyer’s patches (PPs) were shielded, thus specifically labelling intestinal cells. In all experiments with *Kaede* mice, expression of Kaede-red protein was based upon comparison with ambient light-treated surgery control mice (labelled ‘non-converted’ or NC). In PC mice immediately after surgery, ILCs (Fig. 4a,d) and CD4 T cells (Fig. 4b,e) in the small intestine expressed Kaede-red protein compared with NC controls. ILCs within the shielded mLN were not labelled by this process (Fig. 4c). Analysis of the mLN ~24 h after exposure of the intestine to violet light revealed a significant number of Kaede-red^+^ ILCs within this site (Fig. 4f) demonstrating the direct trafficking of these cells from the intestine to the mLN. Gating specifically on the CCR6^+^ ILC3s and CCR6^−^Sca-1^+^ putative ILC2s revealed that both populations had migrated from the intestine (Fig. 4f). While only a modest proportion of CD4 T cells in the mLN of PC mice at 24 h expressed Kaede-red protein (Fig. 4g), substantially more of the ILCs were labelled (Fig. 4h) and this was true of both the ILC3 and ILC2 subsets (Fig. 4i,j). Within the iLN at 24 h post surgery, very few ILCs or T cells expressing Kaede-red were detected (Fig. 4k,l). These data demonstrate that ILC populations in the gut are able to traffic to the draining mLN.

### LTi-like ILC3s require CCR7 to traffic to LNs

**Figure 5:**
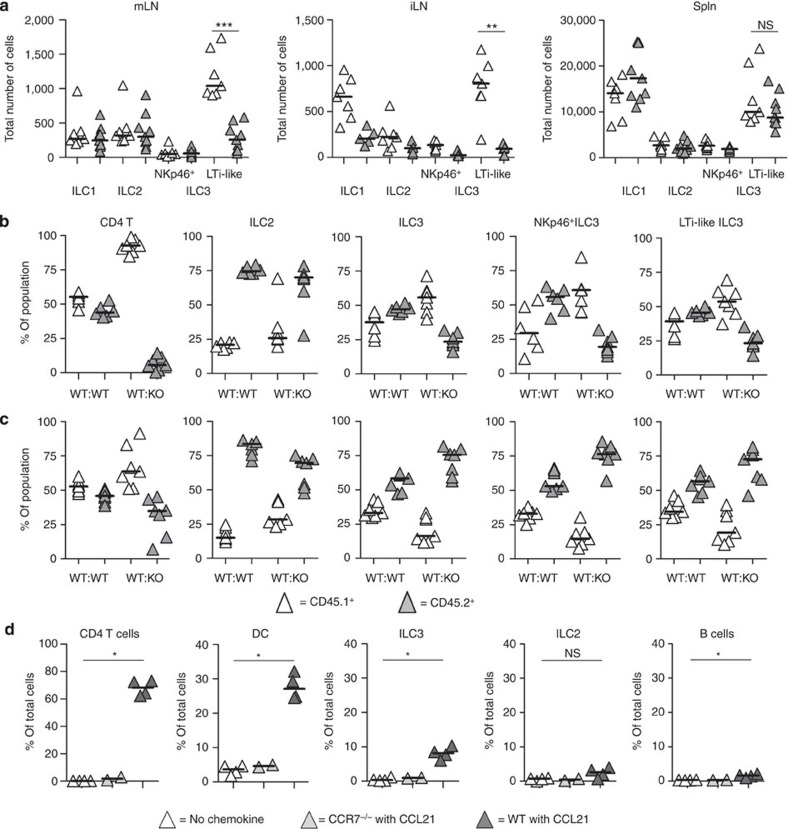
LTi-like ILC3s require CCR7 to reach the mLN. (**a**) Enumeration of the number of ILC1, ILC2, NKp46^+^ and LTi-like ILC3s per individual mLN (*n*=7 and 9), iLN (*n*=7 and 5) or spleen (*n*=7 and 9) of WT and *CCR7*^−/−^ mice. Percentage of CD4 T cells, ILC2s, total ILC3s and NKp46^+^ and LTi-like subsets of ILC3s in the mLN (*n*=6 and 7; **b**) and spleen (*n*=7 and 7; **c**) of bone marrow chimeric mice. Chimeras were generated using CD45.1^+^ CD45.2^+^ hosts, lethally irradiated and reconstituted with CD45.1^+^ WT and either CD45.2^+^ WT (WT:WT) or CD45.2^+^ *CCR7*^−/−^ (WT:KO) bone marrow. Values shown are a percentage of stated non-host cells only, with residual host CD45.1^+^ CD45.2^+^ cells excluded. (**d**) Percentage of CD3^+^ CD4^+^ T cells, CD11c^+^ DCs, Lin^−^ IL-7Rα^+^ CCR6^+^ ILC3, Lin^−^ IL-7Rα^+^ CCR6^−^ ICOS^+^ ILC2 or B220^+^ B cells prepared from WT or *CCR7*^−/−^ mLN that have migrated towards CCL21 in a transmigration assay (*n*=4, 2 and 4). *P<0.05, **P<0.01, ***P<0.001 and ****P<0.0001 (Mann–Whitney non-parametric, two-tailed test). Data are pooled from three (**a**) or two (**b**–**d**) independent experiments, bars represent the median values in all graphs.

Homing of lymphocytes and DCs to LNs requires CCR7 (ref. 29), and both embryonic LTi cells and adult splenic LTi-like cells express *CXCR5* and *CCR7* mRNA^26^,^30^; therefore, we hypothesized that CCR7 was required for trafficking of ILC3s into LNs. In *CCR7*^−/−^ mice, the total numbers of LTi-like ILC3s were significantly reduced in both mLNs and iLNs compared with WT tissues (Fig. 5a and Supplementary Fig. 6). Notably, total numbers of NKp46^+^ ILC3s, as well as both ILC1 and ILC2 populations, were not significantly different in mLNs. Furthermore, no significant difference in the number of LTi-like ILC3s was detected in WT and *CCR7*^−/−^ spleens, indicating that the difference in LNs was not simply due to a systemic reduction in LTi-like ILC3s in *CCR7*^−/−^ mice (Fig. 5a). To investigate whether LN entry of LTi-like ILC3s was dependent upon direct signalling through CCR7, mixed BM chimeras were generated using an ~1:1 mix of either WT (CD45.1):WT (CD45.2) or WT (CD45.1):*CCR7*^−/−^ (CD45.2) BM (Fig. 5b,c and Supplementary Fig. 7). The use of CD45.1^+^ CD45.2^+^ host mice enabled any residual host cells within these chimeras to be identified and excluded from the analysis, thus removing any possible bias from the persistence of radio-resistant host cells. Consistent with a dependency on CCR7 for CD4 T-cell entry into LNs^29^, in WT:*CCR7*^−/−^ chimeras, ~90% of the LN CD4 T cells were of WT BM origin. This bias was not seen in the WT:WT BM chimeras, validating the mixed BM chimera approach. The vast majority of LTi-like ILC3s in the LNs of WT:*CCR7*^−/−^ BM chimeras were derived from WT BM, while this was not the case for ILC2s in LNs (Fig. 5b). The homing of both *CCR7*^−/−^ CD4 T cells and LTi-like ILC3s to the spleen was much less impaired than in LNs, as CCR7 is not critical for entry to this tissue (Fig. 5c). To further test whether ILC3s were functionally sensitive to CCR7 ligands, a transmigration assay using WT mLN cells was performed. ILC3s, but not ILC2s, migrated towards CCL21, albeit less efficiently than CD4 T cells or DCs (Fig. 5d), consistent with functional CCR7 expression. In summary, LTi-like ILC3s, but not other ILC subsets, require CCR7 to enter LNs.

### Accumulation of LTi-like ILC3s post infection requires CCR7

**Figure 6:**
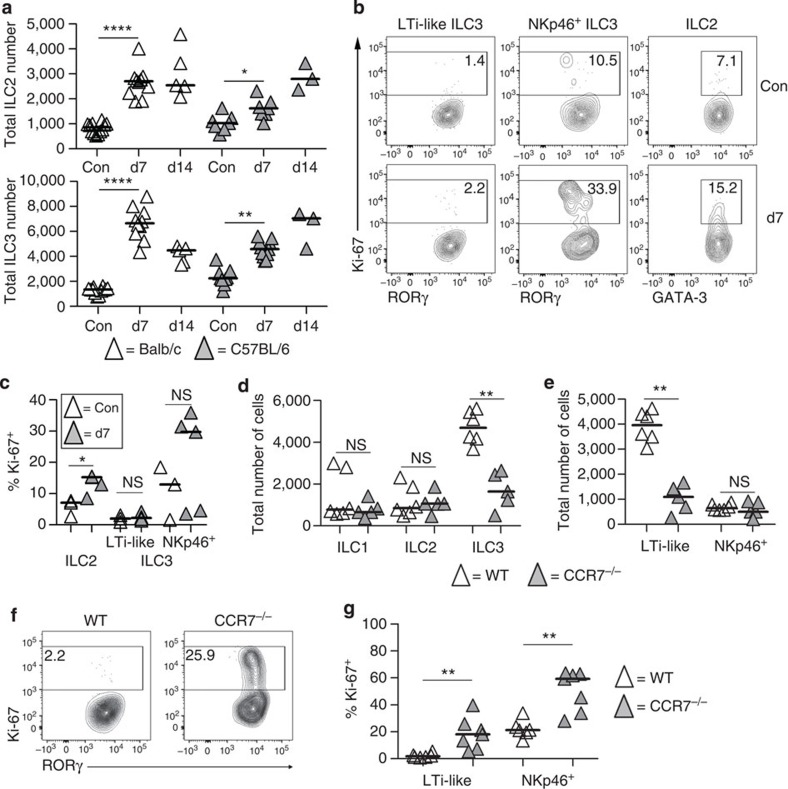
Trafficking of LTi-like ILC3s to the mLN after *H. polygyrus* infection requires CCR7. (**a**) Total numbers of ILC2s and ILC3s per mLN in control versus infected mice, 7 and 14 dpi (*n*=12, 10, 5, 9, 11 and 3). (**b**) Detection of Ki-67 expression among LTi-like ILC3s, NKp46^+^ ILC3s and ILC2s in mLN from control and infected C57BL/6 mice at 7 dpi; data representative of three mice per cell type from two independent experiments. (**c**) Percentage of LTi-like ILC3s, NKp46^+^ ILC3s and ILC2s in the mLN that express Ki-67 in control and infected C57BL/6 mice at 7 dpi (*n*=3 and 5). (**d**) Total numbers of ILC1s, ILC2s and ILC3s per mLN from infected WT and *CCR7*^−/−^ mice at 7 dpi (*n*=6 and 5). (**e**) Total numbers of LTi-like and NKp46^+^ ILC3s per mLN from infected WT and CCR7^−/−^ mice at 7 dpi (*n*=6 and 5). (**f**) Expression of Ki-67 by WT and *CCR7*^−/−^ LTi-like ILC3s in the mLN at 7 dpi, representative of six mice from two independent experiments. (**g**) Percentage of LTi-like and NKp46^+^ ILC3s expressing Ki-67 in the mLN of C57BL/6 WT and *CCR7*^−/−^ mice at 7 dpi (*n*=6 and 7). *P<0.05, **P<0.01, ***P<0.001 and ****P<0.0001 (Mann–Whitney non-parametric, two-tailed test). Data are pooled or representative of two independent experiments, bars represent the median values in all graphs.

Our studies using the *Kaede* mice demonstrated that ILCs, particularly the ILC3s, were able to traffic from the intestine to the mLN, but we sought to further investigate ILC trafficking using an alternative system. To this end, we used an infection model with the natural mouse parasite *Heligmosomoides polygyrus*. Following oral gavage with infective larvae, the helminths penetrate the submucosae of the small intestine, eliciting a strong Th2 response in the draining mLN. Control of this infection involves both innate and adaptive responses with accumulation of ILC2 cells previously reported^31^,^32^. It was also recently shown that *H. polygyrus* infection can induce IL-1β production^33^, dampening IL-25 and IL-33 responses and aiding pathogen chronicity, while within the lung IL-1β signals cause expansion of ILC3s^34^. Consistent with previous studies, in BALB/c mice, where the infection is cleared, we observed a significant increase in ILC2s in the mLN 7 days post infection (dpi; Fig. 6a)^31^,^32^. In contrast, within C57BL/6 mice, which fail to clear the infection, no significant accumulation of ILC2 was observed by 7dpi, although by 14 dpi numbers appeared more comparable to infected BALB/c mice, again consistent with previous observations^31^. Strikingly, in both strains of mice there was also a significant increase in the number of ILC3s within the mLN after infection, likely reflecting IL-1β production and/or intestinal damage caused by the parasite (Fig. 6a). To investigate whether ILC accumulation in the mLN reflected trafficking or *in situ* proliferation, expression of Ki-67 was assessed (Fig. 6b,c). Among ILC populations in the mLN, there was no evidence of proliferation by the LTi-like ILC3s in control or infected mice, while both NKp46^+^ ILC3s and ILC2s showed evidence of increased Ki67 expression after *H. polygyrus* infection, therefore, indicating that ILC3 accumulation in the mLN after *H. polygyrus* infection reflects trafficking of cells from the intestine. This accumulation was dependent upon CCR7, as numbers of ILC3, but not ILC1 or ILC2 populations remained significantly reduced in *CCR7*^−/−^ mLNs 7 dpi compared with C57BL/6 WT (Fig. 6d). Furthermore, this reduction was specific to the LTi-like ILC3 population, since there was no significant difference in the number of NKp46^+^ ILC3s in WT versus *CCR7*^−/−^ mLN at 7 dpi (Fig. 6e). Surprisingly, analysis of Ki-67 expression revealed that in the absence of CCR7, proliferation of both the LTi-like and NKp46^+^ ILC3 populations was substantially increased after infection (Fig. 6f,g), indicating dysregulation of the normal response to this parasite. In summary, these data show that accumulation specifically of LTi-like ILC3s in the draining mLN is dependent upon CCR7.

## Discussion

Here we demonstrate that the interfollicular areas of mucosal draining LNs contain a microenvironment populated by numerous LTi-like ILC3s. Using *Kaede* mice we show that ILC3s are able to traffic to the draining mLN. We report that in the absence of cell-intrinsic CCR7 signalling, the LTi-like ILC3 subset, which forms the majority of ILC3s in LNs, is significantly reduced. *In vitro*, ILC3s are able to migrate towards a ligand of CCR7, CCL21. Finally, using a helminth infection model we show that accumulation of LTi-like ILC3s in the draining LN requires CCR7 expression.

Although a rare population within secondary lymphoid tissue, concentrated clusters of ILC3s can be found within interfollicular areas of LNs. This region is a key site for interactions during the initial stages of adaptive responses, with both antigen-specific CD4 T and B cells specifically located here before migration into the follicle^35^. Given their expression of MHCII, but a lack of CD80 and CD86 (ref. 14), ILC3s are unlikely to drive T-cell proliferation under homeostatic conditions. Rather, these cells likely interact with effector cells after priming, regulatory T cells or memory CD4 T cells recirculating through the tissue. While many more ILC3s were detected within the interfollicular areas of the mLN compared with peripheral LNs, antigen-specific CD4 T cells were increased in the peripheral LNs of *MHCII*^Δ*ILC3*^ mice after peptide immunization, indicating that ILC3s function in a regulatory manner within these tissues as well as within the gut and mLN^14^. Integration of innate stimulatory signals induced by adjuvants or infectious agents may shape the outcome of ILC3:CD4 T-cell interactions^36^, perhaps through inducing expression of co-stimulatory molecules.

While we know that ILC3s are concentrated in the intestine, and now the mLN, the relationship between the populations at these sites was unclear. Our observation that photoconverted ILC3s within the gut of *Kaede* mice can readily traffic from the intestine to the draining mLN leads us to question where does ILC3-mediated control of CD4 T-cell responses to commensal bacteria^14^ actually occur *in vivo*? Is antigen transferred to ILC3 within the mLN from other migrating cells, or could ILC3 potentially acquire antigen in the gut and traffic directly to the mLN? Given both their ability to migrate and their location in LNs, it is possible to propose a model where these cells acquire commensal antigens in the gut and present it to responding CD4 T cells within interfollicular areas. Although deletion of MHCII on ILC3s did not affect their frequency in the mLN^14^, it is not clear whether antigen uptake is required for trafficking from the gut. Further understanding of the specific role of co-stimulatory molecules by ILCs, and whether or not these change following immunization, is required to really understand how these cells might regulate CD4 T cells.

A surprising discovery from the phenotyping of LN ILCs was that a high proportion of ILCs in the mdLN were ILC3s, which, along with the mLN, appears to distinguish it from peripheral LNs, although ILC3s were still found here and also regulated CD4 T-cell numbers. In contrast to the gut, however, there is little evidence of many ILC3s within the lung^16^, arguing against trafficking from this site to the mdLN, perhaps pointing instead to a role in regulating responses to bacterial antigens from the lower respiratory tract. In all LNs analysed, however, it appears that the number of ILC3s is highly CCR7-dependent. This would suggest a common mechanism by which these cells enter LNs, likely through the afferent lymphatics, a CCR7-dependent process based upon comparisons with T cells^37^. It remains possible that ILCs may enter LNs from the circulation through high endothelial venules in a CCR7-dependent manner, however, ILC3s are largely CD62L^−^4. Progenitor populations have been described in the BM and blood^22^,^27^; however, it is not clear that ‘mature’ ILC populations exist in the circulation. Furthermore, we cannot rule out the possibility that the observed reduction in numbers of LTi-like ILC3 in *CCR7*^−/−^ LNs does not, to some extent, result from the additional absence of T cells in these tissues. The partial rescue of *CCR7*-deficient ILC3 numbers in the mesenteric, but not inguinal, LNs of mixed BM chimeras is suggestive of some cell-extrinsic effects in this tissue, which may be especially relevant, given our observation of vastly reduced numbers of ILC3 specifically in the mLN of T-cell-deficient mice. Our data also show that different ILC populations utilize distinct homing receptors for migration. Even among the ILC3s, those expressing NKp46 showed no clear requirement for CCR7 in reaching the mLN, an observation that also applies to ILC2s. On the basis of expression of Ki-67, ILC2s appear more proliferative than ILC3s, and it seems likely that changes in the number of ILC2s reflect a combination of proliferation and trafficking, with data from the *Kaede* mice showing that they are capable of migrating from the intestine to the mLN. It is possible that activation through IL-33 or thymic stromal lymphopoietin (TSLP)^19^,^38^ may be driving migration to the draining LN, with a reliance on alternative chemokine receptors to CCR7.

While the effects of ILC populations on B-cell responses in LNs have not been explored, ILC3s do support T-independent switching to IgA within isolated lymphoid follicles^11^, and it was recently shown that within the spleen, marginal zone B-cell responses are influenced by ILC3s^39^. It is noteworthy in this latter study that the phenotype of the human splenic ILC3 population described was distinct (CD40L^+^NKp46^+^) from the majority of those ILC3s detected in mouse secondary lymphoid tissue. It is also worth noting that DCs migrate through the interfollicular space after arrival in the afferent lymphatics^40^; thus, indirect effects on the adaptive response via effects on DCs may also occur and ILC2s have recently been shown to influence DC migration from the lung to the LN^41^. Previous studies with lymphopenic mice have provided valuable insight into what ILCs can do; however, the challenge now is to understand their roles and contribution within a complete immune system. Since both ILC2 and ILC3 populations share a similar location within LNs and can process and present antigen^14^,^42^,^43^, one can speculate that a balance between different ILC populations may help to shape the outcome of adaptive immune responses. Evidence presented here and in several recent papers indicates that ILC3s play important roles in the control of adaptive immune responses. It will be important to understand exactly how ILCs can influence CD4 T cells and whether we can target this population to enhance or restrict responses.

## Methods

### Mice

Animals were used in accordance with the Home Office guidelines at the University of Birmingham and the University of Glasgow. The following mice were bred and maintained in the Biomedical Services Unit at the University of Birmingham: Balb/c, C57BL/6, *CCR7*^−/−^, *Rag*^−/−^, *TCRα*^−/−^, *ZAP70*^−/−^ (all C57BL/6 background). *Kaede* mice were bred and maintained at the University of Glasgow. For all these experiments, a mixture of male and female mice was used at 6–12 weeks of age. *H2-Ab1*^*fl/fl*^ and *MHCII*^Δ*ILC3*^ mice (all female, aged 8 weeks) were previously described^14^. Genetically modified mouse strains were compared with WT controls from the same facility, while WT mice used in Fig. 1 were obtained from Harlan Laboratories, all female 6–10 weeks of age. Mixed BM chimeras were generated through intravenous transfer of an approximate 1:1 mix of CD45.1^+^ WT and either CD45.2^+^ WT or *CCR7*^−/−^ BM into lethally irradiated CD45.1^+^ CD45.2^+^ WT hosts (2 × 600 rad). *ROR*γ^−/+^ hosts were lethally irradiated (2 × 450 rad) before intravenous transfer of WT or *ROR*γ^−/−^BM. After ~4 weeks, *ROR*γ^−/−^ into *ROR*γ^−/+^ chimeras were additionally treated with anti-Thy1 Abs and then assessed a further 4 weeks later. Control (WT into *RORγ*^−/+^) chimeras were treated with control rat IgG (Sigma-Aldrich).

Mice were infected with ~200 L3 larvae of *H. polygyrus* larvae by oral gavage.

Mice were immunized i.p. with 100 μg 2W1S peptide on D0 and D2, and then taken 6 days later. A pool of peripheral (auxillary, brachial, cervical and inguinal) LNs were compared. WT or *ROR*γ^−/−^ into *ROR*γ^−/+^ chimeras were immunized in the front paw pads with 2 × 10^6^ ActA-deficient *L. monocytogenes-*expressing 2W1S peptide (Lm-2W1S). Bacteria were grown overnight in a shaking 37 °C incubator in Luria–Bertani broth supplemented with 20 μg ml^−1^ chloramphenicol, and then subcultured and grown under the same conditions until the OD_600_=0.1. Bacteria were diluted in PBS for injection.

### Surgical procedures

Under anaesthesia, the small intestine of *Kaede* transgenic mice was exposed following laparotomy with a small midline incision. Two- to three-centimetre sections of tissue were exposed to violet light (395 nm UV LED) for 2–3 min each, while PPs and mLN were protected from exposure using high-density black card. Following replacement of the small intestine, the muscle layer was sutured and the skin closed with surgical clips. Zero-hour controls were taken immediately, while mice in 24-h time point were allowed to recover. The small intestine of control mice was exposed to ambient light. Before surgery, the mice received subcutaneous analgesics Carprofen (Rimadyl, Pfizer) and Buprenorphine (Vetergesic, Reckitt Benckiser Healthcare) at 0.1 ml and 0.15 ml per 100 g, respectively.

### Cell culture

Cells were cultured overnight in RPMI/10% FCS with 10 ng recombinant mouse IL-23, and then Brefeldin A was added to a final concentration of 10 μg ml^−1^ for ~4 h before intracellular cytokine staining.

### Flow cytometry

LNs were teased using fine forceps and digested for 25 min at 37 °C in RPMI with 250 μg ml^−1^ Collagenase-Dispase (Roche) and 25 μg ml^−1^ DNase I before the reaction was stopped with 10 mM EDTA^44^. Digested tissue was then crushed through a 70-μm nylon mesh. Individual mLN numbers were generated by dividing the total cell count by five to account for multiple nodes. Small intestine tissue was cut longitudinally into 0.5-cm sections following the removal of PPs and fat and washed in Hanks’ Balanced Salt Solution (HBSS, Gibco) with 2% FCS. Tissue was incubated twice in HBSS with 2 mM EDTA at 37 °C for 20 min in a shaking incubator, and then digested in complete RPMI with 1 mg ml^−1^ Collagenase VIII (Sigma-Aldrich) at 37 °C for ~15 min, or until tissue was fully digested. Cells were passed through 100 and 40 μm cell strainers. Samples in *Kaede* mouse experiments were stained with an eFluor 780 viability dye in PBS (1:1,000, eBioscience) for 30 min at 4 °C before antibody staining. Staining for 2W1S-specific CD4 T cells was conducted using a phycoerythrin (PE)-conjugated 2W1S:I-A^b^ tetramer for 1 h at room temperature, followed by enrichment using anti-PE MicroBeads and MACS enrichment (Miltenyi Biotech)^13^. Antibody staining was performed at +4 °C for 30 min unless otherwise stated. Antibodies raised against the following mouse antigens were used: B220 (clone RA3-6B2, 1:300 or 1:100, eBioscience), CCR6 (clone 29-2L17, 1:100 or 1:25 Biolegend), CD3 (clone 145-2C11, 1:100, eBioscience), CD4 (clone RM4-5, 1:200, Biolegend), CD5 (clone 53-7.3, 1:100, eBioscience), CD11b (clone M1/70, 1:300 or 1:100, eBioscience), CD11c (clone N418, 1:300 or 1:100, eBioscience), CD45 (clone 30-F11, 1:100, eBioscience), CD45.1 (clone A20, 1:100, eBioscience), CD45.2 (clone 104, 1:100, eBioscience), GATA-3 (clone TWAJ, 1:50, eBioscience), I-Ab (clone M5/114.15.2, 1:500, Biolegend), IL-7Rα (clone A7R34, 1:100, Biolegend, eBioscience), IL-22 (clone 1H8PWSR, 1:50, eBioscience), Ki-67 (clone SolA15, 1:400, eBioscience), NKp46 (clone 29A1.4, 1:100, eBioscience), RORγ (clone AFKJS-9, 1:25, 1:50, eBioscience), Sca-1 (clone D7, 1:200, eBioscience), T-bet (clone eBio4B10, 1:25, eBioscience). The lineage dump channel contained antibodies against B220, CD3, CD5, CD11b and CD11c, except in Figs 1a–e and 4 and Supplementary Figure 1, where CD11b was not included. Intracellular staining was carried out using the FoxP3 fixation and permeabilization kit (eBioscience), or in cases where it was necessary to preserve YFP using Cytofix/Cytoperm Plus (BD Biosciences), both according to the manufacturer’s instructions. Addition of Spherotech Accucount blank particles was used to calculate cell frequencies. Flow cytometry was performed on a Fortessa or LSR II analyzer using the FACSDiva6.2 software (BD Biosciences), with data subsequently analysed with the FlowJo software (Tree Star).

### Transmigration assay

In all, 2 × 10^6^ cells (WT or *CCR7*^−/−^ mLN) in 100 μl RPMI 2% FBS were loaded into transwell inserts with a diameter of 6.5 mm and pore size of 5 μm (Corning Transwell Permeable Supports, 3421, Sigma-Aldrich) and placed in wells containing ±20 nM CCL21 in RPMI with 2% FBS. Plates were incubated for 3 h at 37 °C, and then 5.5 mM EDTA was added to bottom well and plate left on ice for 10 min, before migrated and input cells were analysed by flow cytometry. Percentages of migrated cells shown are as a proportion of the total number of cells retrieved from migrated and input wells. All samples were analysed in triplicate in each experiment, with the exception of *CCR7*^−/−^ controls, which were analysed in duplicate, and average values displayed.

### Immunofluorescence and image analysis

Tissue sections from experimental mice were cut and stained as described previously^13^,^44^. Briefly, 6-μm-thick sections of tissue were cut, fixed in cold acetone at 4 °C for 20 min and then stored at −20 °C before staining. Antibodies raised against the following mouse antigens were used: CD3 (clone eBio500A2, 1:50, eBioscience), CD31 (clone 390, 1:200, eBioscience), CD169 (clone 3D6.112, 1:800, AbD Serotec), F4/80 (clone BM8, 1:50, eBioscience), GATA-3 (clone TWAJ, 1:25, eBioscience), ICOS (clone C398.4A, 1:25, Biolegend), IgM (1:200, Jackson ImmunoResearch), IL-7Rα (clone A7R34, 1:25, eBioscience), KLRG-1 (clone 2F1, 1:50, eBioscience), RANKL (clone IK22/5, 1:50, eBioscience) and RORγ (clone AFKJS-9, 1:30, eBioscience). Detection of RORγ and GATA-3 expression required amplification of the signal as described previously^44^. Purified rat primary antibodies against the transcription factors were detected with donkey anti-rat-IgG-FITC (1:150, Jackson ImmunoResearch), and then rabbit anti-FITC-AF488 (1:200, Life Technologies) and then with donkey anti-rabbit-IgG-AF488 (1:200, Life Technologies). Biotinylated anti-CD3 antibodies were detected with SA-AF555 (1:500, Life Technologies). Sections were counterstained with DAPI (Invitrogen) and mounted using ProLong Gold (Invitrogen). Slides were analysed on a Zeiss 780 Zen microscope (Zeiss). Percentage of RORγ^+^ ILCs in interfollicular areas was quantified using the Zen software (Zeiss).

### Statistics

Data were analysed using the GraphPad Prism (versions 5.01 and 6.04). Sample sizes of atleast four data points were used where possible. Samples were only excluded from analysis where total cell yield isolated from tissue was greatly reduced because of technical error. Non-parametric Mann–Whitney test was used to determine significance, which was set at *P*=<0.05. The median values were calculated and used in all analyses.

## Author contributions

E.C.M. designed, performed and analysed experiments and wrote the paper; S.H. designed, performed and analysed experiments, C.L.M. performed experiments, E.E.H. performed experiments, B.L. designed experiments, V.C. performed experiments, K.J.F. provided reagents for experiments, R.R.M. provided reagents, designed experiments and wrote the paper; M.R.H. performed experiments and analysed data; G.F.S. designed experiments and wrote the paper; S.M. designed experiments, analysed data and wrote the paper; D.R.W. designed, performed and analysed experiments and wrote the paper.

## Additional information

**How to cite this article:** Mackley, E. C. *et al.* CCR7-dependent trafficking of RORγ^+^ ILCs creates a unique microenvironment within mucosal draining lymph nodes. *Nat. Commun.* 6:5862 doi: 10.1038/ncomms6862 (2015).

## Supplementary Material

Supplementary Information Supplementary Figures 1-7.
