# Corrigendum: CCR7-dependent trafficking of RORγ^+^ ILCs creates a unique microenvironment within mucosal draining lymph nodes

**DOI:** 10.1038/ncomms11186

**Published:** 2016-03-29

**Authors:** Emma C. Mackley, Stephanie Houston, Clare L. Marriott, Emily E. Halford, Beth Lucas, Vuk Cerovic, Kara J. Filbey, Rick M. Maizels, Matthew R. Hepworth, Gregory F. Sonnenberg, Simon Milling, David R. Withers

Nature Communications 6: Article number:5862 10.1038/ncomms6862 (2015); Published: 01092015; Updated: 03292016

Previous work by Tomura *et al*. reporting the generation and use of *Kaede* transgenic mice was inadvertently omitted from the reference list of this article and should have been cited at instances where these mice are referred to. For example, in the Results section, Tomura *et al*. should have been cited as follows: ‘Since ILC3s are concentrated within the gut, we sought to test whether an ILC3 bias in the mLN reflected direct trafficking of ILC3s from the intestine to the mLN using transgenic *Kaede* mice (Tomura *et al*.)'. The Methods section should have included the following: ‘The *Kaede* mice used in this study were kindly provided and transferred by Dr Miwa in Tsukuba University and Dr Tomura and Dr Kanagawa in RCAI, RIKEN, Japan.'

Tomura, M., *et al*. Monitoring cellular movement *in vivo* with photoconvertible fluorescence protein ‘Kaede' transgenic mice. *Proc. Natl Acad. Sci. USA*. **105**, 10871–10876 (2008).

