# Supplementary material for: CCR7-dependent trafficking of RORγ+ ILCs creates a unique microenvironment within mucosal draining lymph nodes
Source: Nat Commun. 2015 Jan 9;6:5862. doi: 10.1038/ncomms6862 (PMC4354100; doi:10.1038/ncomms6862)
Supplement: Supplementary Information — Supplementary Figures 1-7. [file ncomms6862-s1.pdf]

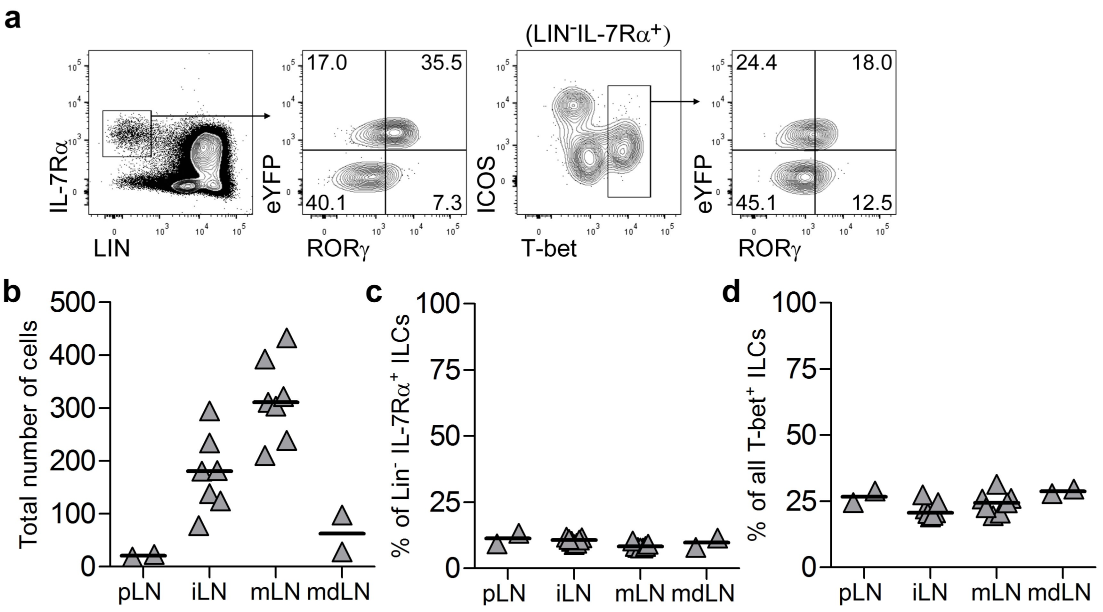

Supplementary Figure 1: LNs of *RORγ cre* x *ROSA26 eYFP* mice. (**a**) Gating strategy to identify ILCs that have turned off RORγ expression ($eYFP^+ROR\gamma^-$). $T\text{-}bet^+$ ILCs were analysed for eYFP and RORγ expression to assess the ILC1 population derived from ILC3 cells. (**b**) Total number of $T\text{-}bet^+eYFP^+ROR\gamma^-$ cells per LN, bars show median. (**c**) Percentage of ILCs that are $T\text{-}bet^+eYFP^+ROR\gamma^-$ in different LNs. (**d**) Percentage of $T\text{-}bet^+$ ILCs that are $eYFP^+ROR\gamma^-$ in different LNs. Data pooled from 2 independent experiments, (n= 2, 7, 7, 2). Bars show median values in all graphs.

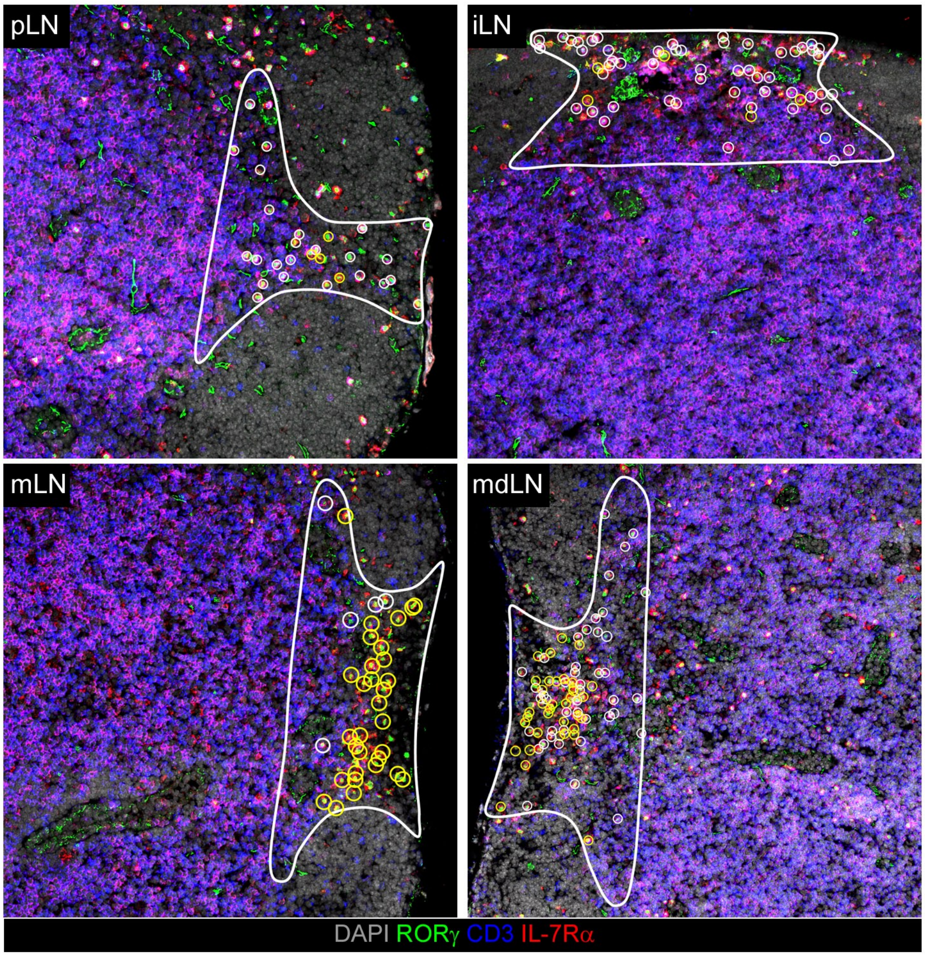

Supplementary Figure 2: Quantitation of ILC3 versus RORγ^+ T cells in interfollicular areas. Images of LNs showing how RORγ^+ cells within the interfollicular areas of pLN, iLN, mLN and mdLN were analysed. The spaces between follicles were marked and $CD3^+ROR\gamma^+IL\text{-}7R\alpha^+$ (white) and $CD3^-ROR\gamma^+IL\text{-}7R\alpha^+$ (yellow) identified and enumerated.

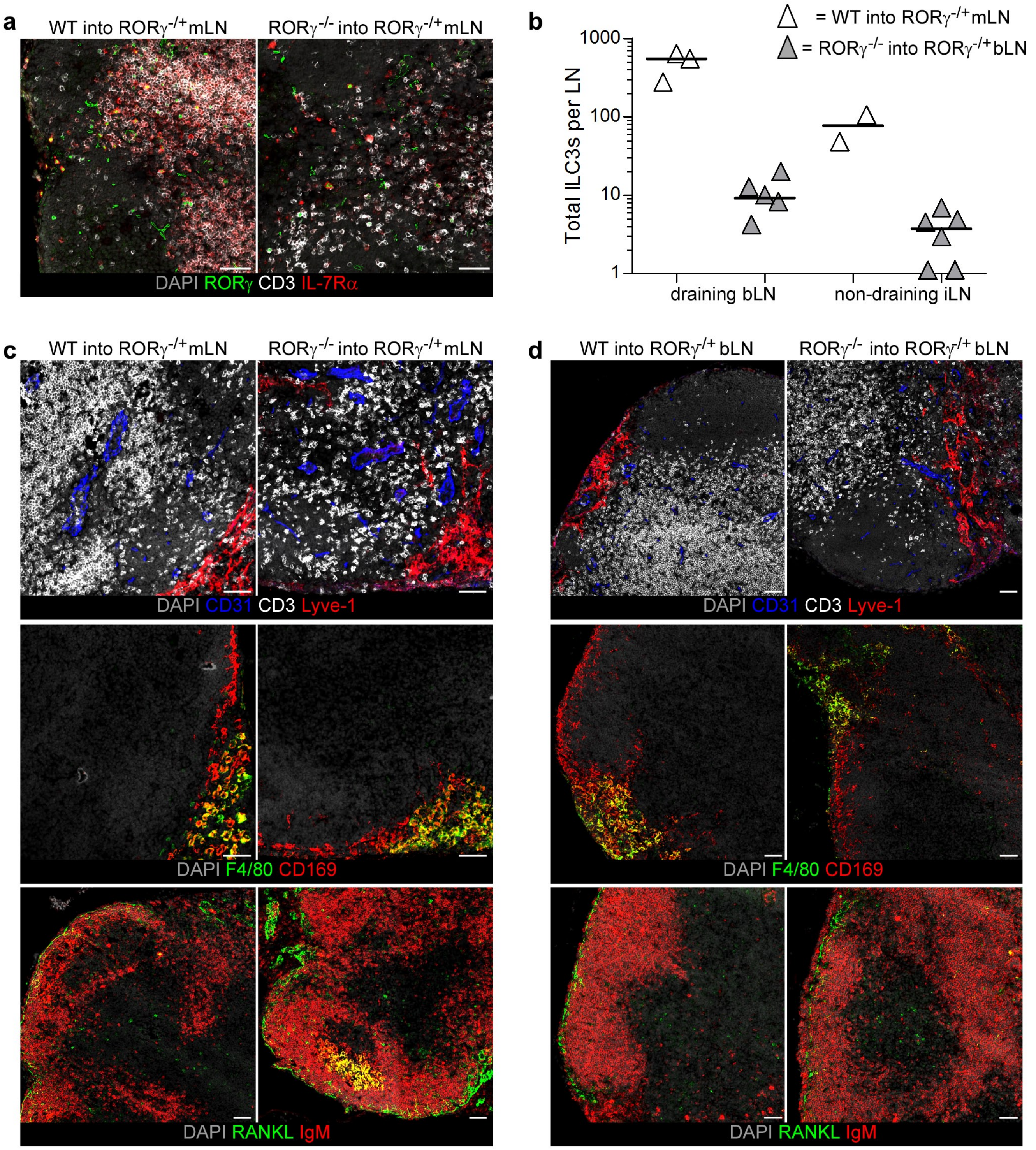

Supplementary Figure 3. Normal lymph node architecture and stromal populations in chimeric mice where ILC3s are depleted. Chimeras were generated through transferring WT or *ROR*g$^{-/-}$ bone marrow into lethally irradiated RORg$^{+/-}$ hosts. (**a**) Expression of RORg, IL-7Rα and lack of CD3 was used to identify ILC3s in the mLN of chimeric mice. (**b**) Numbers of ILC3s in the draining bLN (n= 3, 6) or the inguinal LN (n= 2, 6) of chimeric mice. Mice were immunised subcutaneously at the base of the paw with 2 x 10^6 *L. monocytogenes*-expressing 2W1S. Expression of different stromal and hematopoietic markers in (**c**) the mLN, and (**d**) the draining bLN, of chimeric mice. Scale bar represents 50µm. Data representative of 3 WT: *ROR*g$^{+/-}$ and 6 *ROR*g$^{-/-}$: *ROR*g$^{+/-}$ chimeras from 1 independent experiment.

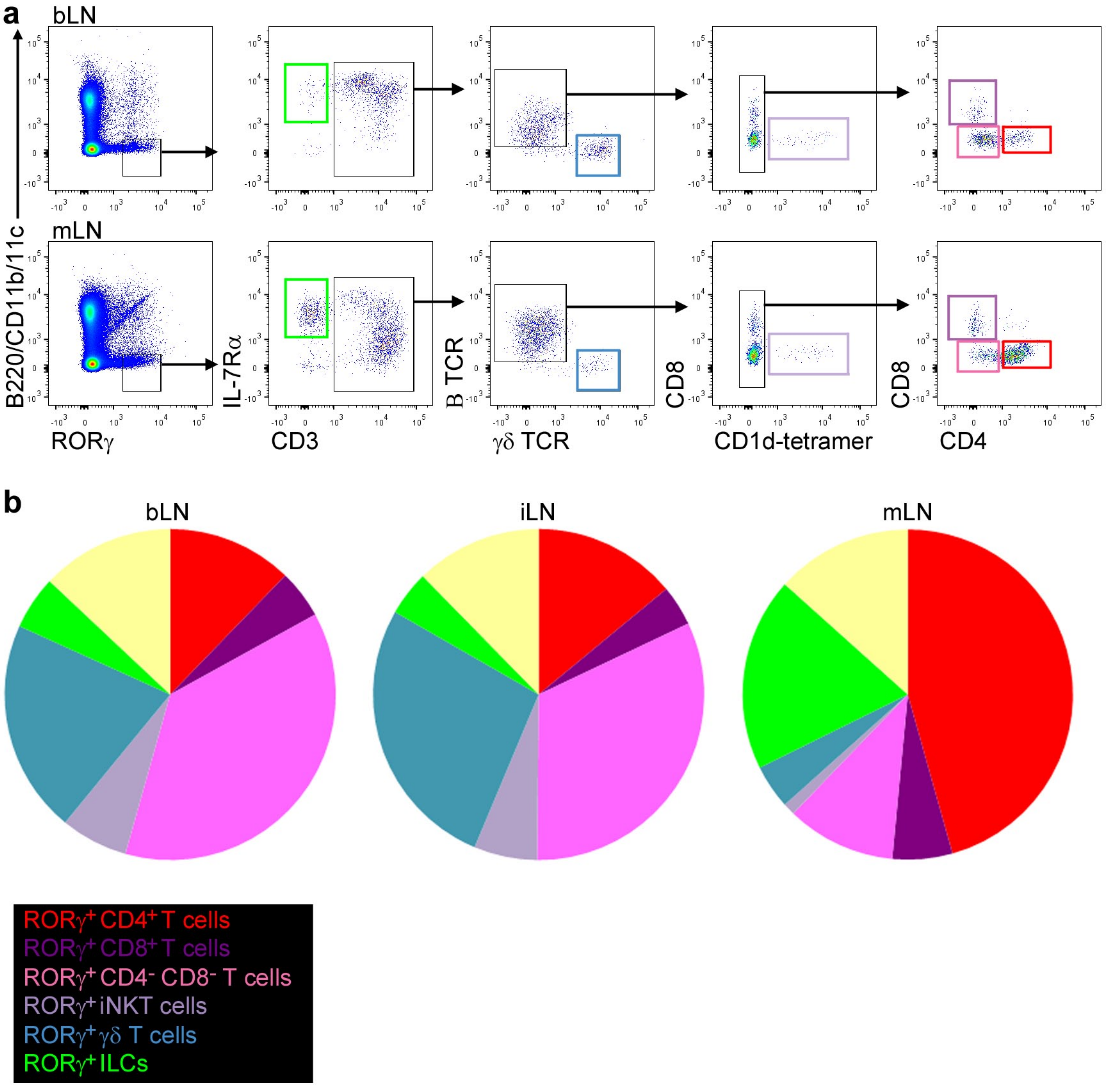

Supplementary Figure 4: Analysis of RORγ^{+} cells within LNs. RORγ^{+}B220^{-}CD11c^{-}CD11b^{-} cells within LNs were analysed by flow cytometry with T cell subsets and ILC3s identified. (**a**) Gating strategy used to identify different RORγ^{+} populations. (**b**) Pie charts showing the average percentage of different RORγ^{+} populations within bLN, iLN and mLN (n = 4 mice per group). Data pooled from two independent experiments.

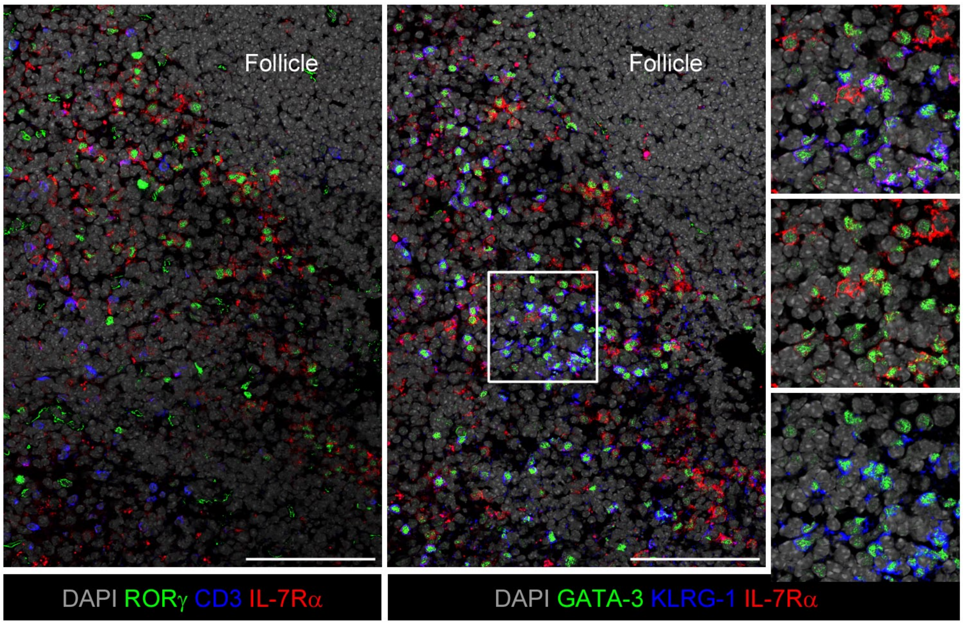

Supplementary Figure 5: Identification of ILC2s and ILC3s in *ZAP70*$^{-/-}$ mLN. Serial sections of *ZAP70*$^{-/-}$ mLN focused on the interfollicular area and stained to identify ILC3s (RORγ^{+}CD3^{-}IL-7Rα^{+}) and ILC2s (GATA-3^{+}KLRG-1^{+}IL-7Rα^{+}). Scale bar shows 100µm. Data representative of 3 mice.

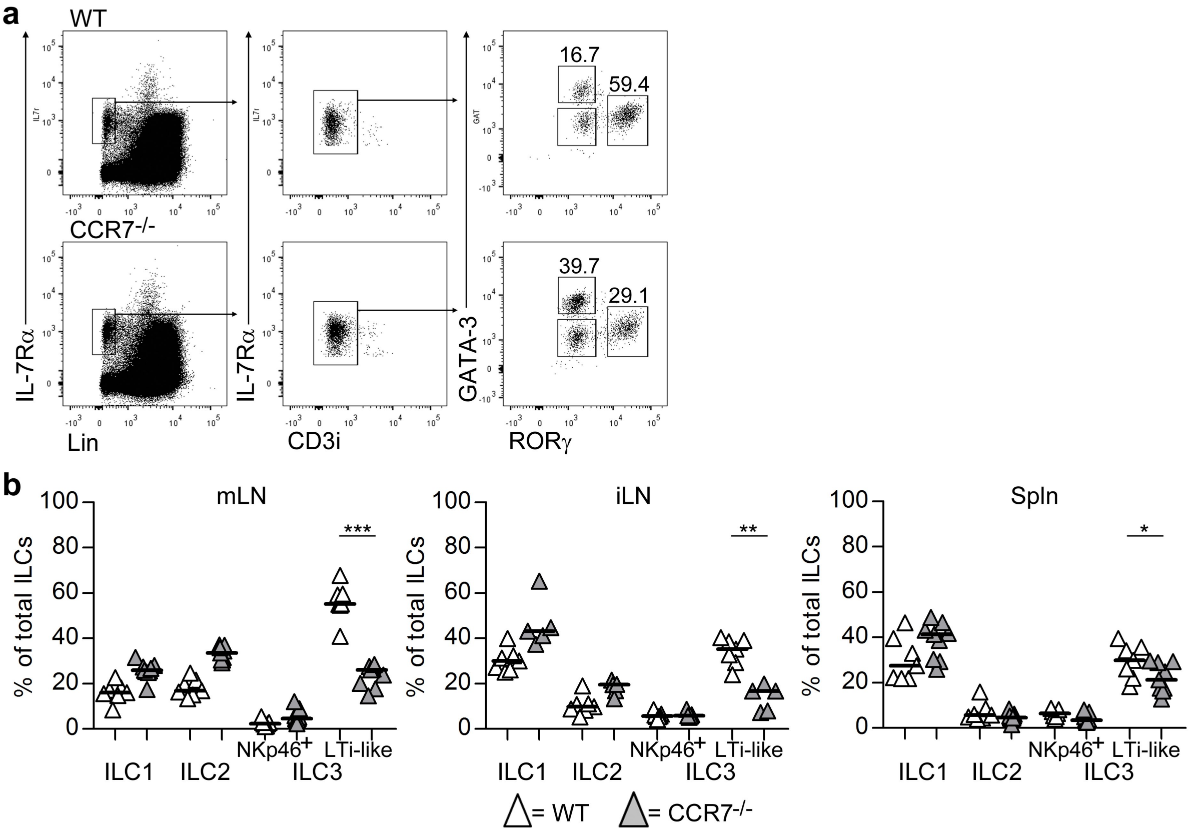

Supplementary Figure 6: Identification of ILCs in *CCR7*$^{-/-}$ tissues. (**a**) Identification of ILC populations in WT and *CCR7*$^{-/-}$ mLN. (**b**) ILC1, ILC2, NKp46^{+} ILC3 and NKp46^{-} ILC3 populations in mLN, iLN and spleen of WT and *CCR7*$^{-/-}$ mice, as a percentage of the Lin^{-}IL-7Rα^{+} population (n = 7, 9 mice for mLN and spleen, 7, 5 for iLN). **P* < 0.05, ***P* < 0.01 and ****P* < 0.001 (Mann-Whitney nonparametric, two-tailed test). Data pooled from three independent experiments, bars show median.

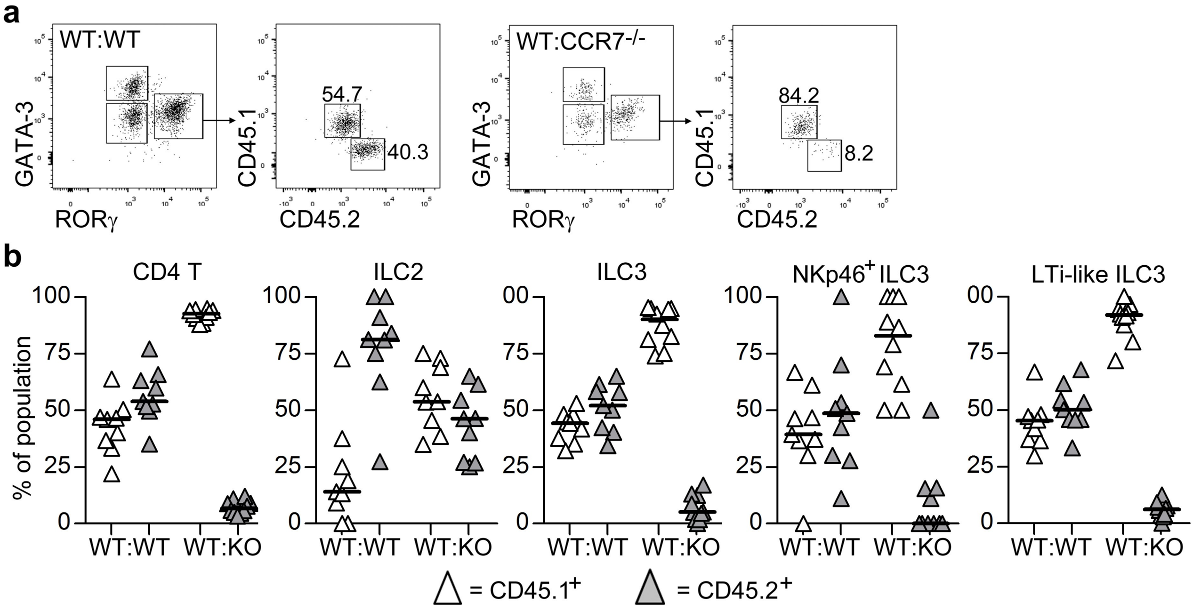

Supplementary Figure 7: Identification of ILCs in WT:*CCR7*$^{-/-}$ mixed bone marrow chimeras. (**a**) Gating strategy for identification of CD45.1^{+} and CD45.2^{+} ILC3s in WT:WT and WT:*CCR7*$^{-/-}$ BM chimeras, as well as persisting CD45.1^{+} CD45.2^{+} host cells. Percentage of cells within gate shown. (**b**) Percentage of CD45.1^{+} or CD45.2^{+} CD4 T cells, ILC2s, ILC3s and NKp46^{+} and LTi-like subsets of ILC3s in the iLN of bone marrow chimeric mice (n= 7, 7). Data pooled from 2 independent experiments. Chimeras were generated using CD45.1^{+} CD45.2^{+} hosts, lethally irradiated and reconstituted with CD45.1^{+} WT and either CD45.2^{+} WT (WT:WT) or CD45.2^{+} CCR7$^{-/-}$ (WT:KO) bone marrow. Values shown are a percentage of stated non-host cells only, with CD45.1^{+} CD45.2^{+} cells excluded.
